# Intelligent Physical Exercise Training in a Workplace Setting Improves Muscle Strength and Musculoskeletal Pain: A Randomized Controlled Trial

**DOI:** 10.1155/2017/7914134

**Published:** 2017-08-07

**Authors:** Tina Dalager, Just Bendix Justesen, Gisela Sjøgaard

**Affiliations:** Department of Sports Science and Clinical Biomechanics, University of Southern Denmark, Odense, Denmark

## Abstract

**Purpose:**

To assess effects of 1-year Intelligent Physical Exercise Training (IPET) on musculoskeletal health.

**Methods:**

Office workers were randomized 1 : 1 to a training group, TG (*N* = 193), or a control group, CG (*N* = 194). TG received 1 h supervised high intensity IPET every week within working hours for 1 year and was recommended to perform 30 min of moderate intensity physical activity for 6 days a week during leisure. The IPET program was based on baseline health measures.

**Results:**

No baseline differences were present. An intention-to-treat analysis showed significant between-group effect for muscle strength but not for musculoskeletal pain. However, a per-protocol analysis of those with an adherence of ≥70% demonstrated a significant between-group effect for neck pain during the past three months. Several significant within-group changes were present, where TG and TG ≥ 70% demonstrated clinically relevant pain reductions whereas minimal reductions were seen for CG.

**Conclusion:**

IPET and recommendations of moderate intensity physical activity demonstrated significant between-group effect on muscle strength. Interestingly, significant within-group reductions in musculoskeletal pain were seen not only in TG but also in CG. This may underlie the lack of such between-group effect and shows that a possible positive side effect of merely drawing attention can improve musculoskeletal health.

## 1. Introduction

During the past decades evidence has emerged that physical activity and fitness are associated with decreased mortality and positive health outcomes [1–4]. Despite this, most adult people in the Western World are insufficiently active, that is, not meeting the international recommendations of moderate to vigorous activity [5, 6]. In addition, due to a technological evolution we are facing an increased sedentary workforce [1] that is illustrated by the fact that as much as 27% of European workers are sitting all or most of the time of a workday [7]. Such lifestyle prompts low muscle strength and cardiorespiratory fitness and has consequences that among others are high prevalence of musculoskeletal pain. In Europe musculoskeletal pain accounts for approx. 40% of all occupational diseases and is considered a growing problem [8]. The presence of musculoskeletal pain has been associated with reduced quality of life for the individual, decreased productivity and increased sickness absence at the workplace, and economic consequences for the society [8–10]. Thus, it is essential to identify strategies that can counteract these potential health problems.

The workplace has been suggested as a specially prioritized arena for health promotion, as it provides an opportunity to reach a large and diverse population and engage individuals who might not otherwise have time and/or face other obstacles to participate in physical activity [11, 12]. Studies have already pinpointed positive effects of workplace interventions promoting health and physical activity on improvements in physical fitness as well as reductions in sickness absenteeism, job stress, and musculoskeletal pain [10, 13–15]. Strong evidence was found for relieving upper extremity musculoskeletal pain by implementing strength training [16].

As the workplace involves a large and diverse population, not all employees may benefit from the same training program. Despite the same occupational exposure, there are individual differences regarding physical capacity and health issues that also need to be accounted for in a health promoting physical exercise training program. Therefore, we have developed a physical activity concept termed Intelligent Physical Exercise Training (IPET). For each employee at the workplace, we have designed individually tailored physical exercise training by balancing the occupational exposure with the individual's physical capacity and health risk indicators [17]. The training regimen combines various forms of physical exercise training to improve cardiorespiratory fitness, individual health risk indicators, and musculoskeletal health based on relevant baseline health check.

The aim of the present paper was to investigate effects on musculoskeletal health. Changes in muscle strength and musculoskeletal pain were monitored after a one-year intervention with one weekly hour of supervised high intensity IPET at the workplace combined with recommendations of leisure time physical activity. Based on a number of earlier findings [14] we tested the one-sided hypotheses that muscle strength increased and pain decreased with this physical exercise training intervention.

## 2. Materials and Methods

### 2.1. Study Design

The present paper presents secondary analysis of a randomized single-blinded parallel controlled trial conducted in Denmark from May 2011 to March 2014. Primary outcome analysis, one-year change in cardiorespiratory fitness, has been published previously and demonstrated, for example, a significant increase in maximal oxygen uptake [18]. The protocol for this study has been presented in detail regarding recruitment procedure and outcome measures [17]. In short, office workers were recruited from six different companies located across Denmark: two private companies, two public municipalities, and two national boards. The enrolment was sequential in six strata from May 2011 to March 2012 with baseline, one-year, and 2-year follow-up measurements.

Participants were assigned an arbitrary ID number by an authorized member of the technical staff to ensure allocation concealment. When all the participants had completed baseline measurements, they were individually randomized within each company using the identification number and a random number computer algorithm.

Due to the content of the intervention, physical exercise training, participants, instructors supervising the IPET intervention, and health ambassadors could not be blinded to group allocation. The examiners performing the health checks were blinded to each participant's group allocation and at follow-up testing, the participants were told not to tell the examiners the group to which they were allocated. All test personnel and investigators involved in data treatment were blinded to the randomization.

All participants were informed about the purpose and content of the project and gave written informed consent to participate in the study. The study was conducted in accordance with the CONSORT statement [19] and conformed to The Declaration of Helsinki and approved by the Local Ethical Committee of Southern Denmark (S-20110051). The study qualified for registration in ClinicalTrials.gov (NCT01366950).

### 2.2. Subject Recruitment

**Figure 1:**
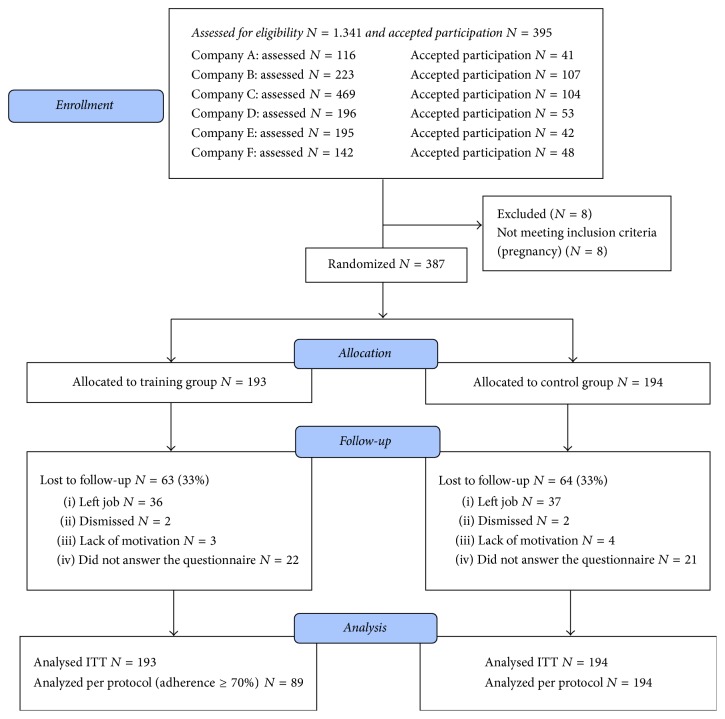
Flow-chart, updated from Sjøgaard et al. 2014 [17].

Office workers who worked ≥25 h per week within an office environment were eligible and were invited by e-mail containing a link to an Internet-based questionnaire regarding working conditions, health behavior, musculoskeletal pain, and physical activity level. Exclusion criteria were (a) cardiovascular disease, chest pain during physical exercise, myocardial infarction (lifetime history), stroke, severe musculoskeletal disorders, symptomatic herniated disc, and other severe disorders of the spine, postoperative conditions, or lifetime history of severe trauma and (b) pregnancy. Exclusion was based on questionnaire replies and baseline health check. A total of 1.343 employees were invited; 395 accepted the invitation and were assessed for eligibility. Eight females were excluded due to pregnancy and a total of 387 participants were randomized to either TG (*n* = 193) or CG (*n* = 194). See Figure 1 for flow of participants.

### 2.3. Intervention

The participants in CG received no workplace physical exercise training or other information regarding recommended leisure time physical activity but were encouraged to maintain their lifestyle as usual. The participants in TG were to follow the training intervention that was based on the theoretical framework of IPET. Each participant in TG received an individually tailored exercise training program based on outcome measures of the baseline health check and questionnaire data [17].

In short, the exercise training program was performed during working hours, at or near the workplace. The program lasted one hour a week for 2 years, the first year was fully supervised, and, during the second year, monthly supervision of a weekly training session was provided. The present paper only presents one-year effects.

Strength training was included based on measures of baseline muscle strength, balance test, core and neck/shoulder stability, and pain intensity in specified body regions. For each measure, cut-off points were identified to allocate individual training modes, duration, and intensity [17].

For each training session, 10 min was allowed for getting to and from the training area. The first 20 min was for all participants allocated to cardiorespiratory fitness training, including 10 min warm-up, due to office workers' sedentary working condition. Hereafter, for the last 30 min each participant trained his or her specific exercises according to the individual training program provided. The individualized IPET programs were composed following the guidelines from the American College of Sports Medicine [6], as well as specific strength training exercises for the neck and shoulder region [20, 21]. Participants performed 3 sets of 8 repetitions with an intensity of 60–80% of one repetition maximum, though for neck and shoulder exercises intensity was to pain limits or as heavy as possible with proper technical execution. In total, 32 individual training programs were developed, of which nine covered more than 85% of the participants' needs, most of which included neck/shoulder strength training and extra cardiorespiratory training. Examples of exercises for strength training were for neck and shoulders: shrugs, reverse flies, 1-arm row, and lateral raise. For large muscle groups: bench press, lunges, squat, and pelvic lift. For low back and core stability: basic and side plank, back extension, and diagonal raise. The exercises could vary depending on the available equipment or individual preferences, but the chosen exercise targeted the specific muscle group. The instructor, who was a sports science based exercise training specialist, assessed training intensity for each participant at the end of every training session using the Borg scale (Rating of Perceived Exertion (RPE 6–20)) [22]. Target training intensity was RPE 14–17 for every training session.

In addition to the workplace intervention, participants in TG were encouraged by health ambassadors to engage in moderate physical activity (64–76% HR max, RPE 12-13) for six days a week during leisure time or a minimum of three hours weekly. Health ambassadors were appointed for every 10–15 employees by the company's middle managers. The appointed health ambassadors participated in the training at the workplace but were not included in the randomized TG.

### 2.4. Data Collection

All measurements at baseline were performed before the randomization and repeated after one and two years. Besides demographics and information on weight, height, body mass index, and body fat%, the following health variables constituted the data for the present paper.

#### 2.4.1. Muscle Strength

Maximal isometric muscle strength was measured with Bofors MODEL dynamometer (Bofors Elektronik, Karlskoga, Sweden) mounted in a reproducible standardized setup for four tests: back extension, abdominal flexion, shoulder elevation, and arm abduction [23]. In short, for back extension and abdominal flexion, the participant was standing in an upright position, with relaxed arms, and with a strap attached to a strain gauge dynamometer around the shoulders at the level of deltoid insertion. The pelvis was placed against a plate with the upper edge aligned with the iliac crest. The participant was instructed to tighten the core muscles and with maximal strength bend backward and forward, respectively. For shoulder elevation and arm abduction, the participant was seated in a standardized chair with the back vertical and no floor contact with the feet. The head was positioned anatomically neutral. For shoulder elevation two force dynamometers were placed bilaterally above the shoulders one cm medially to the lateral edge of the acromion, and the participant was instructed to elevate the shoulders with maximal strength. For shoulder abduction, elbows were bent 90 degrees and two force dynamometers were placed bilaterally proximal to the lateral epicondyle, and the participant was instructed to abduct the upper arms with maximal strength. In every test the participant completed three maximal voluntary contractions (MVC) with at least a 30 s break between tests (if the 3rd MVC was 5% higher than the first or second test the participant was instructed to perform another test with a maximum of five tests). The highest value was registered and reported in Newton (N) [23].

#### 2.4.2. Musculoskeletal Pain

Musculoskeletal pain in lower back, upper back, neck, and shoulders was measured using the validated Nordic Musculoskeletal Questionnaire [24]. For each site, participants were asked “how many days have you experienced pain in your [body part] during the last three months?” Response categories were (1) 0 days, (2) 1–7 days, (3) 8–30 days, (4) >30 days, or (5) every day. In addition, the participants rated their pain intensity, “on average, how intense was your pain in [name of body part] during the past three months/past seven days?,” on a 10-point numerical box scale ranging from 0 (no pain) to 9 (worst possible pain) [24].

A pain index of the past three months, ranging from 0 to 100%, was calculated for all subjects not lost to follow-up. Additionally 11 participants were excluded from this analysis because not all the required questions for the index calculation were answered, resulting in a total of 261 subjects included (130 for TG, 131 for CG, and 75 for TG ≥ 70%). The pain index was calculated as average normalized values of the four above-mentioned body regions, as the sum of intensity and duration with equal weight. For duration, answers were recorded as follows: 0 days = 0, 1–7 days = 4, 8–30 days = 19, >30 days = 60, and every day = 90. Zero equals 0% and 90 equals 50%. For intensity, 0 on the scale of 0–9 equals 0% and 9 equals 50% [25].

### 2.5. Adherence

Attendance to the weekly supervised training sessions at the workplace for the TG was recorded by the instructor and applied to calculate adherence, defined as the number of attended training sessions out of possible training sessions within the one-year intervention. For a per-protocol analysis, we defined an inclusion criterion to be an adherence in the training sessions performed at the workplace of ≥70% [26].

### 2.6. Statistics

The statistical analyses were based on an intention-to-treat (ITT) approach using STATA version 14. Missing values in either baseline or follow-up measurements were substituted with data carried forward or backward. When measurements had missing values in both baseline and follow-up measurements, these were replaced by means of each respective group. Nonparametric testing was applied, as data did not meet the assumption of normal distribution. Differences in the baseline characteristics were examined by either a Chi square test or Mann–Whitney test depending on type of data. Analyses of intervention effects were performed using the Mann–Whitney test on delta values (follow-up values minus baseline values). Within-group changes were analyzed using the Wilcoxon signed rank test. In addition, a per-protocol analysis (PP) was performed for those participants in TG who had an adherence of minimum 70% (TG ≥ 70%) and including all participants in CG. ITT and PP analyses were performed both for the group of all participants and for the group of participants being a pain case at baseline (intensity ≥ 3). With a logistic regression model, we also analyzed changes in symptom status in terms of the proportion of participants who was a “no pain case” at follow-up, adjusted for baseline status. The statistical significance level was set to 0.05. Tests of one-sided hypotheses were deemed significant if a two-sided *P* value was less than 0.1.

## 3. Results

### 3.1. Baseline Characteristics

At baseline, there were no differences in demographics between TG and CG or between TG ≥ 70% and CG (Table 1). Mean ± SD for age was 44 ± 10 years, 74% were females, and participants had an average body mass index of 25.4 ± 5.1 kg/m^2^. No differences were present for muscle strength. For musculoskeletal pain symptoms, there were no differences except for a small difference in low back pain in the past seven days between TG and CG.

### 3.2. Intervention Effects

At follow-up, 28% in TG and 30% in CG were lost to follow-up (Figure 1). The overall adherence for TG was 56 ± 29% corresponding to 29.2 training sessions. No difference between companies or between sexes regarding adherence was present. The 89 participants in TG ≥ 70% had a mean adherence of 80 ± 8% corresponding to 41.7 training sessions. In total, 77% of the participants in the TG were offered specific exercises for the neck and shoulders (ranging from 10 to 20 minutes) and 65% were offered core stability exercises (ranging from 5 to 20 minutes). One hundred and four participants (54%) were allocated to both types of training.

The ITT analysis showed statistically significant larger changes in TG for muscle strength compared with CG. Changes are shown as delta values in Table 2. No significant differences in changes were present between TG and CG for any of the musculoskeletal pain variables. Likewise, when comparing pain cases, no significant differences in changes were present between TG and CG (Table 3).

In the PP analysis, TG ≥ 70% demonstrated statistically significant larger increases in muscle strength compared with CG (Table 2). In addition, TG ≥ 70% improved neck pain in the past three months significantly compared with CG. Likewise, pain cases among TG ≥ 70% significantly improved neck pain in the past three months and left shoulder pain in the past three months and seven days compared with CG (Table 3).

Additionally, within-group changes occurred. TG significantly increased muscle strength except for right arm abduction strength and decreased musculoskeletal pain in all body regions (Table 2). CG significantly increased left shoulder elevation strength but decreased muscle strength for right and left arm abduction strength, and no changes were observed for the other muscle strength outcomes. Furthermore, CG significantly decreased musculoskeletal pain for all body regions. Further, TG ≥ 70% significantly increased muscle strength except for right arm abduction strength and decreased musculoskeletal pain. Finally, pain cases decreased musculoskeletal pain in all body regions for each of the three groups: TG, TG ≥ 70%, and CG.

The logistic regression model did not demonstrate any significant differences in change of symptoms status between TG and CG or between TG ≥ 70% and CG.

The analysis of pain index in the past three months conducted on a subsample of 261 participants showed no differences between groups at baseline with an overall pain index of 19.0 ± 18.3. The pain index change in TG (−8.0 ± 13.8) was not statistically significantly different from that in CG (−5.5 ± 13.5), and likewise there was no difference between TG ≥ 70% (−8.4 ± 14.2) and CG.

## 4. Discussion

The major finding of this study was the significantly increased muscle strength among office workers after one year of workplace health promotion including IPET. Surprisingly, the significant increases in muscle strength were in the ITT analysis not accompanied by a significant between-group effect in musculoskeletal pain (TG versus CG). Only in the PP analysis, where adherence to the intervention was 70% or more, was a significant between-group effect (TG ≥ 70% versus CG) found for neck pain in the past three months. In addition, the pain case group also significantly decreased neck pain in the past three months as well as left shoulder pain in the past three months and the past seven days compared to CG. Several within-group changes were observed. TG and TG ≥ 70% significantly increased muscle strength, whereas CG decreased muscle strength for arm abduction. Musculoskeletal pain decreased significantly not only within TG and TG ≥ 70% but also within CG.

The lack of a significant between-group effect in musculoskeletal pain is contradictory to previous studies demonstrating clinically relevant between-group effect in musculoskeletal pain following workplace physical exercise training interventions [20, 27, 28]. Office workers' occupational exposure implies extensive inactivity for the large muscle groups that impacts on cardiovascular fitness. Therefore, the present study intervention included a minimum of 20 minutes of the allocated one hour weekly training to high intensity aerobic exercise training [18]. Depending on the individual workers' capacity and health profile, specific strength training exercises of the painful body regions were allocated for 5–20 minutes in the training program. Specific strength training of the neck and shoulder region for 20 minutes was only allocated to 46% of TG, and 20 minutes of core stability training was allocated to even fewer [17]. Thus, the accumulated weekly training volume for the strength training exercises may not have been sufficient to result in significant differences in pain reductions between TG and CG. Previously we have demonstrated a significant dose-response relationship between training volume and change in pain [25]. In the present study, measures were not taken to quantify training volume per session but the increases in muscle strength in this study correspond to only approx. a 3% increase for TG. This is half the size or even less than other studies have reported in which significant between-group effect for musculoskeletal pain has been demonstrated [29–31]. This may explain that only between-group effects were shown for those with high adherence (TG ≥ 70%) who demonstrated muscle strength increases of approx. 5%. In addition to insufficient training volume a plausible reason for no significant between-group effect for musculoskeletal pain is training intensity. Studies have shown the importance of a high training intensity rather than high training volume with regard to decreasing musculoskeletal pain [32, 33]. A single training set to failure instead of multiple training sets may also promote adherence.

Of note in the present study is that 50% of CG increased the number of active days at leisure time per week as reported in our previous paper [18]. A reason for this finding may be the high risk of contamination from TG to CG due to randomization being performed on the individual worker-level and not as in previous studies on a department cluster level. This contamination may be the cause of the significant reductions in pain seen in CG, since a previous study showed that also all-round physical exercise was beneficial for decreasing musculoskeletal pain [34]. Although the positive effect within CG explains the lack of effect between groups, it should be noted that TG and TG ≥ 70% demonstrated clinically relevant reductions of >1 whereas this was not the case for CG.

Existing literature suggests that the effectiveness of workplace health promotion interventions is determined by intervention characteristics. Larger effect sizes have been found for multicomponent interventions, where interventions were implemented during paid working hours, and had employee facilitators, and the interventions offered weekly contact [12, 13, 35, 36]. The present study encompassed these four characteristics and showed the positive side effects of also improving CG, thus not demonstrating the expected between-group effects. This shows that merely drawing attention to health aspects at the workplace can improve perceived musculoskeletal health.

### 4.1. Strengths and Limitations

A strength of this study was the high external validity due to mean age and gender distribution of the participants being similar to office workers in the Danish workforce and the companies being located in different parts of Denmark with both private and public sectors being represented. Also, adherence was reported objectively by instructor observation, which was regarded a strength of this study as compared to self-reporting.

A limitation was the low acceptance rate of roughly 30% among the invited employees and unfortunately we do not know the characteristics of those who did not accept to participate. Thus, a potential limitation of this study and reason for not finding between-group effect on musculoskeletal pain could be that it was not attractive to people with severe musculoskeletal pain. Also the low adherence of 56% must be considered as a limitation. In addition, randomization was performed on a worker-level with a high risk of contamination from TG to CG.

## 5. Conclusion

This study demonstrated that one hour of supervised individually tailored physical exercise training once a week integrated into the workday, combined with the recommendation of 30 minutes of moderate physical activity for six days a week, had a significant positive between-group effect on muscle strength. Only participants adhering 70% or more to the worksite training reached significant between-group effect on neck pain in the past three months. Significant within-group reductions of musculoskeletal pain in several body regions were seen for TG, TG ≥ 70%, and CG. The combined evidence from the present and numerous previous studies suggests that it is now “time to move ahead” and implement IPET at a large scale on the labour market. The implementation strategies will be crucial; they are pertinent to systematize, and evidence for best practice is to be documented.

## 6. Future Perspectives: Time to Move Ahead

IPET as an individually tailored training program showed significant effects on muscle strength and cardiorespiratory fitness [18], significant increases in productivity and workability, and a decrease in neck pain and short term sickness absenteeism for TG ≥ 70% [37]. Though IPET in the ITT analysis did not show between-group effect on musculoskeletal pain, the overall results of IPET underline the effectiveness of such an intervention on several relevant health outcomes that are directly linked to all-cause mortality.

Future perspectives for IPET include the development of a framework that corporates also the organizational supports. Physical exercise plays a central role, not only for prevention, but also for treatment of several health problems, yet a large proportion of the population remains inactive. A qualitative study of motivation and barriers to physical exercise at the workplace emphasizes the importance of interaction between management at the workplace, the employees, and the intervention, since management can result in both a facilitation and a barrier [38]. Companies' internal working culture is crucial for the success of future workplace interventions and there is a need for a clear connection between the implementational intentions of the management and the actual implementation. To avoid low acceptance rates and adherence it is important to ensure the legitimacy of the intervention among the managers, participants, and colleagues, as well as centrally organize, structure, and ensure flexibility for all employees during the workday to allow time for physical exercise training [38–41].

Moreover, we need to work with the motivational aspect. Emphasis on physiologically effective exercises in a scientific intervention is not sufficient, we need to implement varying, motivating, and entertaining exercises as it is far more important for adherence and sustained participation [38].

IPET addresses the individual as well as the organization. The interaction between the individual and the environment seems to be a stronger predictor for participation and adherence than individual factors alone. With knowledge within exercise physiology and a focus on the social and psychological factors, a future corporate framework of IPET with organizational support is envisaged to significantly increase the low acceptance rate as well as adherence, to attain high effectiveness of work place physical exercise training studies.

## Figures and Tables

**Table 1:** Baseline characteristics. Data are shown as mean ± SD except for proportion of females. 3 mt = 3 months. 7 d = 7 days. TG = training group, TG ≥ 70% = participants in TG with an adherence of ≥70%, and CG = control group. ^∧^ = significant difference between groups.

	TG (*n* = 193)	TG ≥ 70% (*n* = 89)	CG (*n* = 194)	TG versus CG	TG ≥ 70% versus CG
*Demographics*					
Females (%)	73.1	69.7	74.7	0.729	0.388
Age (years)	44.0 ± 11.0	45.0 ± 11.0	45.0 ± 10.0	0.265	0.863
Height (cm)	171.0 ± 8.9	171.3 ± 9.1	170.3 ± 8.5	0.429	0.398
Weight (kg)	74.1 ± 16.1	74.7 ± 16.8	74.2 ± 17.1	0.834	0.723
Fat (%)	28.9 ± 8.9	28.8 ± 8.6	29.3 ± 8.8	0.646	0.647
BMI (kg/m^2^)	25.3 ± 5.0	25.4 ± 4.7	25.5 ± 5.2	0.759	0.989
*Muscle strength*					
Back extension (N)	534.3 ± 166.0	552.6 ± 166.3	535.8 ± 165.4	0.941	0.407
Abdominal flexion (N)	460.1 ± 145.0	473.0 ± 145.9	461.2 ± 151.1	0.958	0.479
Right shoulder elevation (N)	492.0 ± 163.4	504.8 ± 161.4	491.4 ± 187.6	0.421	0.336
Left shoulder elevation (N)	479.7 ± 164.4	500.5 ± 171.0	472.1 ± 184.2	0.631	0.632
Right arm abduction (N)	249.4 ± 95.4	254.1 ± 98.1	248.7 ± 109.5	0.298	0.140
Left arm abduction (N)	241.1 ± 96.4	245.0 ± 99.5	242.9 ± 110.4	0.225	0.077
*Musculoskeletal pain (scale from 0 to 9)*					
Neck 3 mt	2.7 ± 2.4	2.7 ± 2.4	2.6 ± 2.3	0.958	0.918
Neck 7 d	2.0 ± 2.3	2.1 ± 2.4	2.0 ± 2.2	0.879	0.840
Right shoulder 3 mt	2.1 ± 2.6	2.2 ± 2.6	1.8 ± 2.0	0.994	0.722
Right shoulder 7 d	1.6 ± 2.4	1.6 ± 2.4	1.4 ± 1.8	0.284	0.401
Left shoulder 3 mt	1.2 ± 2.1	1.0 ± 2.0	1.3 ± 1.9	0.065	0.061
Left shoulder 7 d	1.0 ± 2.0	0.9 ± 1.8	1.1 ± 1.7	0.069	0.078
Upper back 3 mt	1.7 ± 2.3	1.7 ± 2.4	1.5 ± 2.1	0.501	0.554
Upper back 7 d	1.3 ± 2.0	1.4 ± 2.2	1.1 ± 1.9	0.583	0.603
Low back 3 mt	2.6 ± 2.5	2.5 ± 2.6	2.1 ± 2.1	0.125	0.410
Low back 7 d	2.0 ± 2.3	2.0 ± 2.4	1.5 ± 1.9	**0.033** ^∧^	0.228

**Table 2:** Delta values (follow-up values minus baseline values). Data are shown as mean ± SD. 3 mt = 3 months. 7 d = 7 days. TG = training group, TG ≥ 70% = participants in TG with an adherence of 70%, and CG = control group. *∗* = significant between groups' effect (test of one-sided hypothesis). § = significant within group effect.

	TG (*n* = 193)	TG ≥ 70% (*n* = 89)	CG (*n* = 194)	TG versus CG	TG ≥ 70% versus CG
*Muscle strength*					
Back extension (N)	8.1 ± 68.4^§^	14.2 ± 80.1^§^	0.7 ± 68.0	0.090^**∗**^	0.064^**∗**^
Abdominal flexion (N)	13.2 ± 48.8^§^	17.6 ± 46.9^§^	1.2 ± 49.9	0.012^**∗**^	0.005^**∗**^
Right shoulder elevation (N)	15.6 ± 63.9^§^	18.5 ± 66.0^§^	4.2 ± 74.9	0.063^**∗**^	0.068^**∗**^
Left shoulder elevation (N)	14.3 ± 62.4^§^	15.5 ± 71.0^§^	6.6 ± 69.2^§^	0.056^**∗**^	0.091^**∗**^
Right arm abduction (N)	−0.4 ± 46.0	1.5 ± 46.0	−6.6 ± 49.8^§^	0.024^**∗**^	0.029^**∗**^
Left arm abduction (N)	4.4 ± 51.7^§^	7.6 ± 57.3^§^	−4.2 ± 50.9^§^	0.001^**∗**^	0.001^**∗**^
*Musculoskeletal pain (scale from 0 to 9)*					
Neck 3 mt	−1.0 ± 2.1^§^	−1.2 ± 1.9^§^	−0.8 ± 1.8^§^	0,449	0,078^**∗**^
Neck 7 d	−0.9 ± 2.0^§^	−1.1 ± 2.1^§^	−0.7 ± 1.8^§^	0,272	0,150
Right shoulder 3 mt	−0.8 ± 2.1^§^	−0.7 ± 2.4^§^	−0.7 ± 2.1^§^	0,556	0,891
Right shoulder 7 d	−0.7 ± 1.8^§^	−0.5 ± 1.9^§^	−0.7 ± 1.7^§^	0,194	0,216
Left shoulder 3 mt	−0.3 ± 1.6^§^	−0.6 ± 1.7^§^	−0.3 ± 1.9^§^	0,956	0,494
Left shoulder 7 d	−0.4 ± 1.5^§^	−0.5 ± 1.5^§^	−0.3 ± 1.6^§^	0,399	0,966
Upper back 3 mt	−0.9 ± 1.8^§^	−1.1 ± 21^§^	−0.7 ± 1.7^§^	0,335	0,298
Upper back 7 d	−0.7 ± 1.7^§^	−0.9 ± 2.2^§^	−0.6 ± 1.4^§^	0,659	0,565
Low back 3 mt	−0.6 ± 2.0^§^	−0.9 ± 2.1^§^	−0.6 ± 2.1^§^	0,932	0,155
Low back 7 d	−0.7 ± 1.9^§^	−0.9 ± 1.9^§^	−0.5 ± 1.8^§^	0,436	0,122

**Table 3:** Baseline and delta values (follow-up values minus baseline values) for pain cases (participants who at baseline had a pain intensity ≥3 for the respective body regions). Data are shown as mean ± SD. 3 mt = 3 months. 7 d = 7 days. TG = training group, TG ≥ 70% = participants in TG with an adherence of 70%, and CG = control group. Musculoskeletal pain (scale from 0 to 9). *∗* = significant between groups' effect (test of one-sided hypothesis). § = significant within group effect. ^∧^ = significant difference between TG and CG at baseline.

	TG			TG ≥ 70%			CG			*P* values on delta values	
	*n*	Baseline	Delta	*n*	Baseline	Delta	*n*	Baseline	Delta	TG versus CG	TG ≥ 70% versus CG
Neck 3 mt	80	5.0 ± 1.8	−2.0 ± 2.6^§^	37	5.1 ± 1.6	−2.4 ± 2.2^§^	84	4.7 ± 1.6	−1.4 ± 2.1^§^	0.133	0.017^**∗**^
Neck 7 d	56	5.0 ± 1.9	−2.5 ± 2.5^§^	29	5.0 ± 2.0	−2.7 ± 2.6^§^	54	5.0 ± 1.8	−2.1 ± 2.4^§^	0.422	0.348
Right shoulder 3 mt^∧^	64	5.2 ± 1.8	−2.5 ± 2.5^§^	33	5.1 ± 1.9	−2.3 ± 2.5^§^	58	4.5 ± 1.5	−2.3 ± 2.3^§^	0.664	0.980
Right shoulder 7 d^∧^	44	5.4 ± 1.9	−2.6 ± 2.9^§^	22	5.3 ± 2.0	−2.2 ± 3.0^§^	40	4.4 ± 1.7	−2.5 ± 2.3^§^	0.712	0.747
Left shoulder 3 mt	35	5.1 ± 2.0	−2.0 ± 2.9^§^	16	4.9 ± 1.7	−3.2 ± 2.2^§^	40	4.3 ± 1.7	−1.7 ± 2.5^§^	0.384	0.026^**∗**^
Left shoulder 7 d^∧^	24	5.7 ± 1.9	−2.9 ± 2.6^§^	11	5.0 ± 1.6	−3.4 ± 2.2^§^	29	4.4 ± 1.8	−2.1 ± 2.3^§^	0.245	0.095^**∗**^
Upper back 3 mt	56	4.8 ± 1.8	−2.7 ± 2.3^§^	26	5.0 ± 1.7	−3.3 ± 2.5^§^	44	4.8 ± 1.7	−2.4 ± 2.5^§^	0.667	0.146
Upper back 7 d	35	5.1 ± 1.9	−3.1 ± 2.5^§^	19	5.1 ± 1.9	−3.8 ± 2.6^§^	29	5.0 ± 2.0	−2.4 ± 2.3^§^	0.427	0.149
Low back 3 mt	87	4.8 ± 1.8	−1.5 ± 2.2^§^	39	4.9 ± 2.0	−2.1 ± 1.9^§^	75	4.3 ± 1.5	−1.7 ± 2.2^§^	0.482	0.296
Low back 7 d	57	4.9 ± 1.9	−2.0 ± 2.4^§^	24	5.3 ± 2.0	−2.6 ± 2.5^§^	39	4.5 ± 1.8	−1.8 ± 2.6^§^	0.429	0.173
